# Gut Microbiota of Young Children Living in Four Brazilian Cities

**DOI:** 10.3389/fped.2020.573815

**Published:** 2020-12-07

**Authors:** Charmaine Chew, Karina Vieira Barros, Virginia Resende Silva Weffort, Hélcio de Sousa Maranhão, Marisa Laranjeira, Jan Knol, Guus Roeselers, Mauro Batista de Morais

**Affiliations:** ^1^Danone Nutricia Research, Singapore, Singapore; ^2^Laboratory of Microbiology, Wageningen University, Wageningen, Netherlands; ^3^Danone Nutricia, São Paulo, Brazil; ^4^Department of Pediatrics, Universidade Federal Do Triângulo Mineiro, Uberaba, Brazil; ^5^Department of Pediatrics, Universidade Federal Do Rio Grande Do Norte, Natal, Brazil; ^6^Faculdade De Medicina Do ABC, São Paulo, Brazil; ^7^Danone Nutricia Research, Utrecht, Netherlands; ^8^Escola Paulista de Medicina, Universidade Federal de São Paulo, São Paulo, Brazil

**Keywords:** gut microbiome, gut microbiota, young children, toddler, preschool

## Abstract

Recent studies have demonstrated that gut microbiota development is influenced by human biogeographic factors such as race, ethnicity, diet, lifestyle or culture-specific variations, and other environmental influences. However, biogeographic variation in gut microbiota assembly remains largely unexplored in Latin America. In this paper, we compared food recall information and microbiota composition of toddlers living in geographically separated urban populations within four states of Brazil. 16S RNA gene sequencing revealed that alpha diversity was similar between the four different populations. Gut microbiota compositions were dominated by members of the phyla Firmicutes and Bacteroidetes, resembling a more adult-like microbiota as compared with those of Western European toddlers of similar age. These findings suggest that inter-individual and nutrition-induced differences were apparent in the fecal microbiota. We conclude that urban dietary pattern plays a larger role in influencing the gut microbiota composition than do biogeographic factors.

## Introduction

As interest in the gut microbiome has rapidly increased in the last decade, attention has turned to the role of early life microbiota development in later life health (1–3). It is now widely recognized that the gut microbiome plays an important role in human metabolic, immunity, and even cognitive development. Nevertheless, the ecological succession and function of the gut microbiome during childhood are not completely understood, as research mainly focuses on infants. Although it has been suggested that the gut microbiome reaches a relatively stable, adult-like state after the first 1–3 years of life (4–6), there are other studies that suggest that its composition continues to develop into the teenage years (7–9).

Early childhood is the most critical phase in various aspects of human development. Factors such as nutrition, environmental changes, and infection can influence the maturation of the immune system. This in turn may severely impact a child's resilience to pathogenic infections. The microbiota plays critical roles in the maturation and priming of the human innate and adaptive immune systems, while the immune system reciprocally orchestrates the stability and function of the host–microbiota symbiosis. However, poor nutrition can directly and indirectly compromise immune function and subsequent susceptibility to infection (10).

Several studies have investigated the nutritional status and feeding patterns of Brazilian children over a broad age range (11–13). The most prevalent form of deficiencies identified were incidences of low vitamin and micronutrient status (14). This observation has urged medical professionals and policymakers to better engage and educate parents and caregivers on the importance of establishing good eating habits that will in the long term benefit their children's health (15).

The gut microbiota also act as a protective physical barrier to pathogenic bacteria, harvest energy from food, and are crucial to synthesis of metabolites required for cellular functions (16–18). Many research groups have focused their investigation on factors such as mode of delivery, type of feeding, and environmental impacts in the 1st year of life (19–22). After weaning, the child is exposed to greater range of food, which slowly evolves the gut microbiota toward adult-like profile.

A report by De Filippo et al. (23, 24) suggests that sanitation and increased amounts of readily accessible acellular nutrients typical for a modern Western-type diet and lifestyle might be key to the selection of microbial dynamics and functionality, which differentiates urban and rural living. Traditionally, the main source of food comes from unprocessed agriculture crops, and progressive urbanization and industrialization have increased accessibility to processed foods. This transition to modern lifestyles has been associated with reduced intestinal diversity and changing microbiota (25). A study that evaluated Brazilian data from the National Demographic and Health Survey (2006–2007) reported that the diet of children in developed urbanized areas consists of higher processed foods and more foods not recommended for their age, like sweets and soft drinks (soda), as compared with children from rural areas. This is particularly prominent in regions such as South, Southeastern, and Midwest Brazil relative to the North and Northeast.

In this cross-sectional cohort study, we conduct a descriptive analysis of microbiota composition and dietary intake by simple food recall information in healthy, young children aged 1–3 years (*n* = 99) within four urban cities in four states in Western Brazil.

## Materials and Methods

### Subjects and Study Population

This cross-sectional observational multicenter study included a total of 99 children aged between 1 and 3 years, enrolled full time in day care centers from different regions of Brazil [Santo André (SAN), State of São Paulo; Uberaba (UBE), State of Minas Gerais; Porto Alegre (POA), State of Rio Grande do Sul; and Natal (NAT), State of Rio Grande do Norte]. Subjects with missing data or no fecal samples were excluded. A 2-day recall food frequency questionnaire (FFQ) was used to collect information on health and diet of the children, and non-compulsory fecal samples were collected for intestinal microbiota composition and function analyses. Participants with prior chronic diseases and congenital malformations or who received antibiotics <31 days prior to sample collection were excluded. Participants' samples that were collected without traceable subject information (e.g., geographical origin) and/or no fecal sample collected at all were excluded from the subsequent analyses. Full written informed consent was obtained from all included participants parents prior to collection of biological samples and any personal information. This study was approved by the ethics committee (CEP-FMABC, Comitê de Ética, Santo Andre) and registered at ClinicalTrials.gov (November 1, 2016; NCT02950740).

### Sample Collection and DNA Extraction

Fecal samples were collected at a single cross-sectional time point from subjects in day care centers in the four cities and frozen immediately at 4°C. Samples were then transported in insulated storage containers and stored at −80°C at São Paulo in GC-2 company laboratory until further analysis.

Samples were processed using QIAGEN DNA Stool Mini-Kit + RNase step (QIAGEN, Hilden, Germany), modified with an additional bead-beating step. Approximately 200 mg of 0.1-mm glass beads were added to 200-mg stool sample and resuspended. Samples were processed using QIAGEN DNA Stool Mini-Kit + RNase step (QIAGEN, Hilden, Germany), modified with an additional bead-beating step. Approximately 200 mg of 0.1-mm glass beads were added to 200-mg stool sample and resuspended in QIAGEN ASL buffer; bead-beating was done using FastPrep24 (M.P. Biomedicals, USA) for three repetitions of 1-min bead-beating with 5-min incubation on ice. Samples were then heated at 95°C for 15 min before centrifugation at 20,000 × g for 1 min. The supernatant was transferred into a clean tube containing an InhibitEX tablet and vortexed to mix proceeding with manufacturer instructions. Isolated fecal DNA was eluded in 50 μl of AE buffer, quantified using a NanoDrop 2000 (Thermo Scientific, DE, USA), and stored at −20°C before used for further analyses. The remaining fecal samples were resuspended in phosphate-buffered saline (PBS) for short-chain fatty acid (SCFA) analysis.

### 16S rDNA Sequencing and Bioinformatics Analysis

From the purified fecal DNA extracts, the V3–V4 hypervariable regions of the bacterial 16S rRNA gene were amplified, using universal primers S-D-Bact-0341-b-S-17 (forward 5′-CCTACGGGNGGCWGCAG-3′) and S-D-Bact-0785-a-A-21 (reverse 5′-GACTACHVGGGTATCTAATCC-3′) (44). Sequencing was performed by LifeSequencing S.L. (Valencia, Spain) on an Illumina MiSeq instrument (San Diego, California, USA).

An adaptation of the “Quantitative Insights Into Microbial Ecology” (QIIME) v1.9.0 package (16) was used to analyze the sequence data (26). Briefly, sequences were clustered into Operational Taxonomic Units (OTUs) based on 97% sequence identity as proxy for bacterial species using VSEARCH v2.03 with exclusion of chimeric sequences identified against the RDP gold database. Taxonomic assignment will be performed using the RDP classifier (27) against the SILVA119 database (28). The microbial species diversity (α-diversity) was calculated using Faith's phylogenetic diversity (PD) (29); and the Shannon index for diversity was calculated by applying correction for the differences in sequencing depths by rarefaction. Sequences are deposited in EMBL-EBI European Nucleotide Archive (Accession number: PRJEB39067).

### Short-Chain Fatty Acid Analyses

SCFA analysis was performed as described by Bakker-Zierikzee et al. (30). Briefly, SCFAs were extracted from fecal PBS homogenate. The homogenates were mixed in formic acid, ethyl butyric acid (1.25 g/L; Sigma USA), and MilliQ water. Some fecal SCFAs such as acetic, propionic, *n*-butyric, *iso*-butyric, *n*-valeric, and *iso*-valeric acids were detected through gas chromatography with capillary column (Varian 3800, Varian Inc., Walnut Creek, CA, USA).

### Statistics Analysis

All statistical analyses were performed using PRISM GraphPad 6 (version 6.02). Samples analyses were done with pairwise Mann–Whitney non-parametric comparison. The interaction of the intestinal microbiota composition in relation to geographical regions was visualized spatially using distance matrices on principal component analysis (PCA), which is an unconstrained ordination method, using CANOCO 5 (2012) software.

## Results

### Subject Background Information

**Figure 1:**
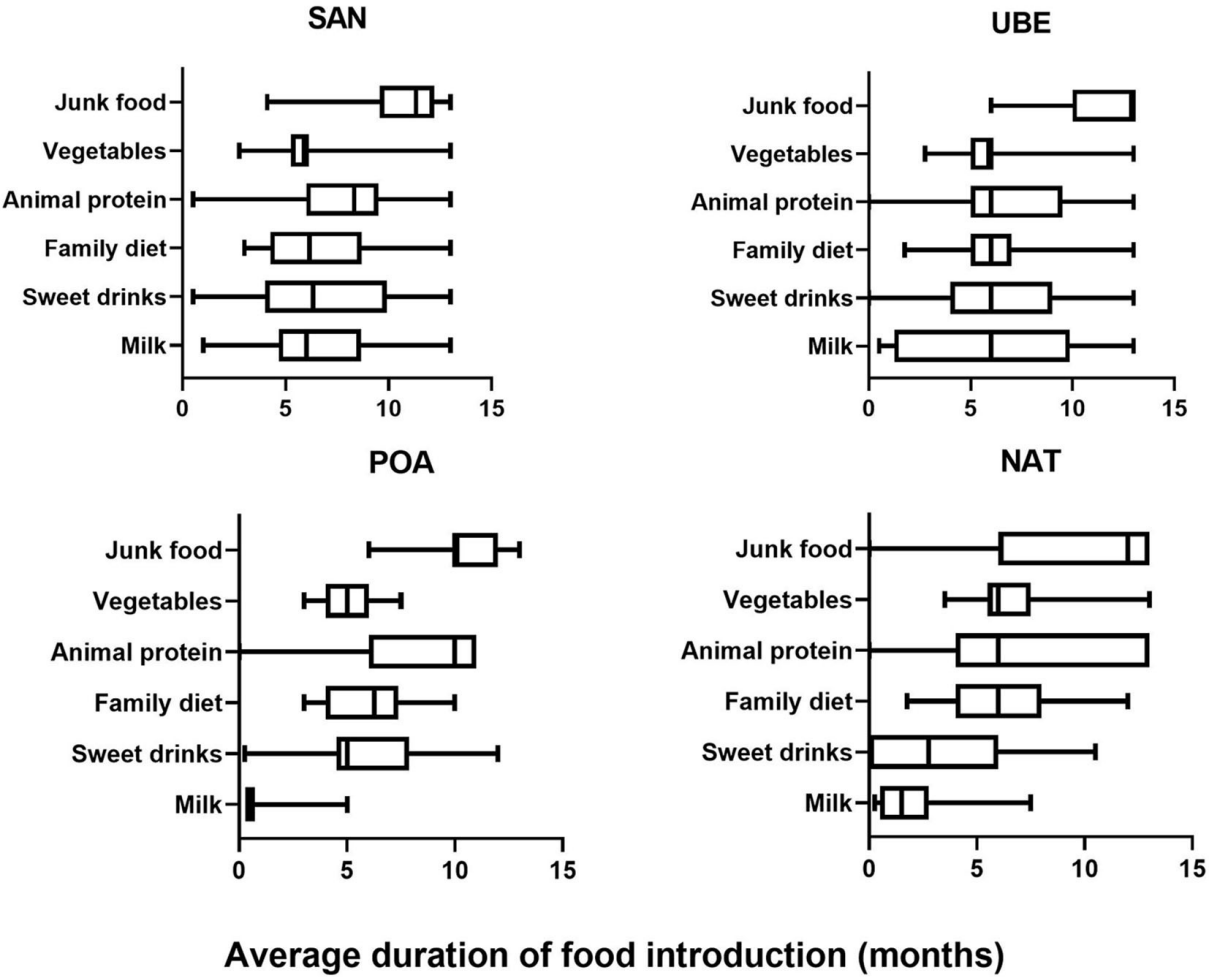
Food diary average frequency consumed by child collected using 2-day food frequency questionnaire (FFQ) across the four cities (SAN, Santo Andre; UBE, Uberaba; POA, Porto Alegre; and NAT, Natal) showing that junk food consumption is prevalent in all four cites after the age of 1 year, while milk consumption varied.

**Figure 2:**
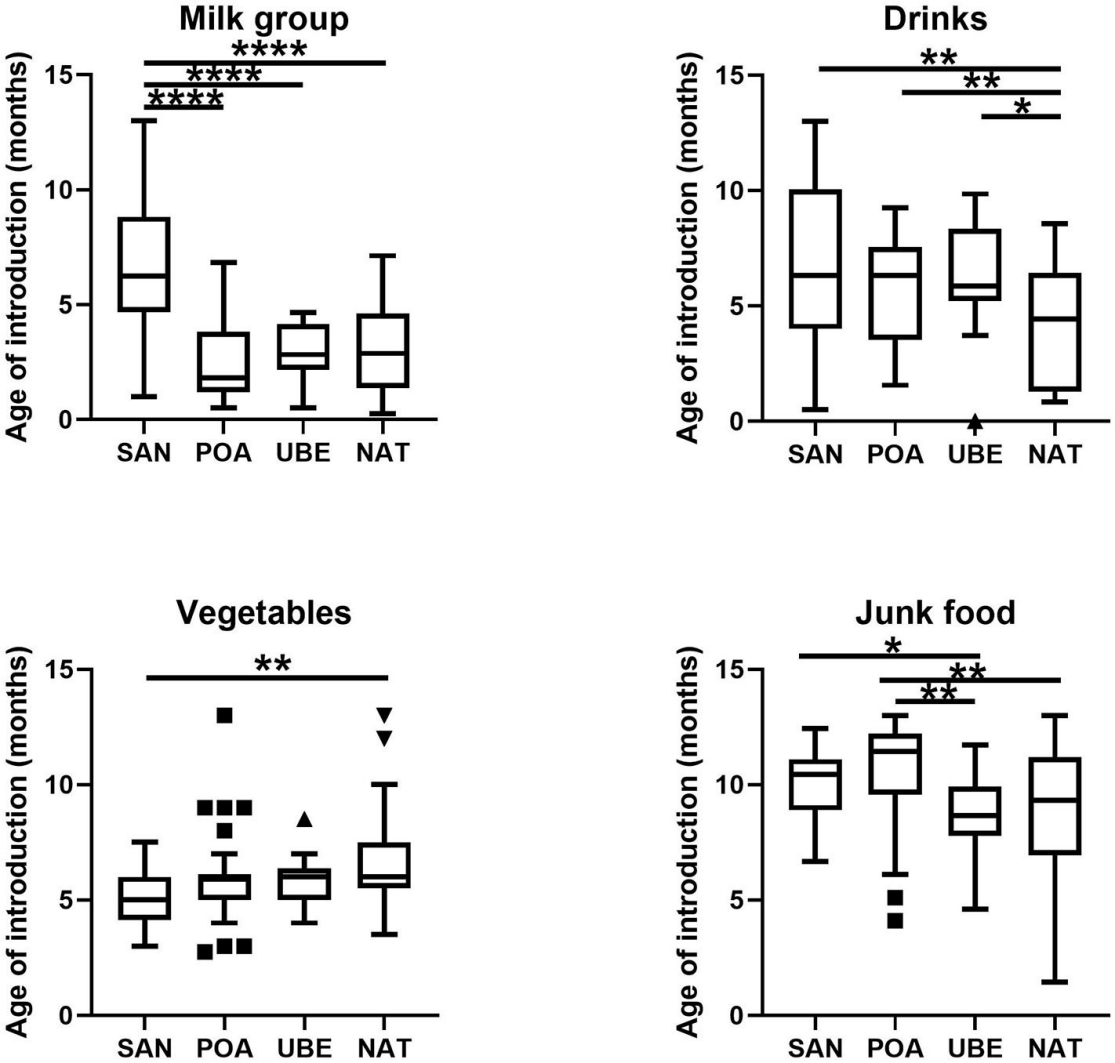
Food groups based on 2-day food frequency questionnaire (FFQ) documented. Milk group includes cow's milk, breastmilk (if any), formula, and yogurt; drinks include sweetened juices and beverages; vegetables are inclusive of legumes; junk food includes sweets, biscuits, and chocolate. Differences in frequencies of complementary foods consumption, such as food puree and family diet, were not shown, as they were not statistically significant. Where *p < 0.05, **p < 0.005, ****p < 0.0001.

From the 116 included participants recruited from public day care centers from four different geographical regions Santo André (*n* = 35) (Southeast region, SAN), Natal (*n* = 31) (Northeast region, NAT), Porto Alegre (*n* = 16) (Southern region, POA), and Uberaba (*n* = 17) (Southeast region, UBE), 99 met all requirements for subsequent analyses. Children in this cohort are evenly distributed in gender (Supplementary Table 1). Based on data collected from 2-day food recall questionnaires, we split each field into five categories: milk (inclusive of cow's milk/formula), vegetables, drinks (excluding water), animal proteins, and junk food (Figure 1). SAN has shown significant later age introduction of milk consumption (*p* < 0.001). All four cities show a high level of junk food introduction before a child is 1 year old, and POA was significantly later than the other cities (Figure 2).

### Microbiota Profiling in Brazilian Children

Initial processing and filtering of raw 16S rRNA gene sequence data resulted in minimum 25,546 high-quality reads per sample, and rarefaction analyses showed slopes reaching a plateau after a rapid initial increase, indicating a sufficient sequencing depth (Table 1). Diversity and richness of the fecal microbiota composition were the highest in participants from Uberaba. There are significant differences between Uberaba and Natal (*p* = 0.012) and Porto Alegre (*p* = 0.0056). Similar results were obtained by other diversity indices (Shannon, Simpson, and Chao1).

**Table 1:** Number of subjects and sequencing statistics by geographical region and Firmicutes/Bacteroidets ratio.

**Location**	**Average reads**	**Minimum**	**Maximum**	**Standard deviation of reads**	**F/B ratio**	**No of samples**
NAT	47,030.42	25,546	285,780	51,838.74	1.81	31
SAN	43,592.63	32,747	55,217	6,843.1	1.47	16
UBE	42,234.69	28,183	54,078	6,501.26	2.0	35
POA	43,909.47	30,632	96,912	14,996.89	1.89	17

**Figure 3:**
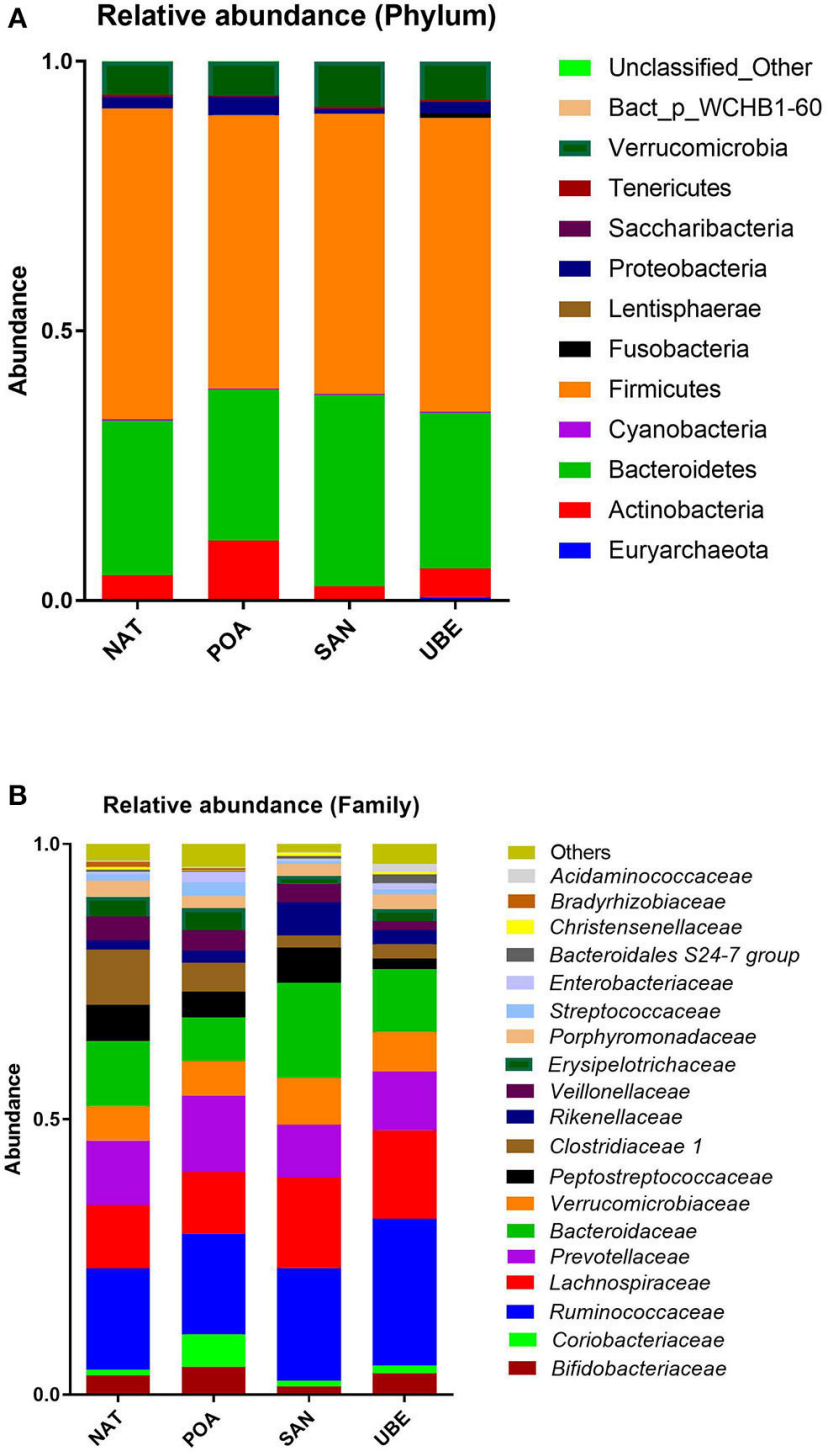
Relative abundance at the **(A)** phylum and **(B)** family level shows dominance of taxa affiliated to the phyla Firmicutes and Bacteroidetes (shown in gray and light blue, respectively).

Taxonomic classification of high-quality sequence data showed that children aged between 1 and 3 years have microbiota dominated by taxa affiliated to the bacterial phyla Firmicutes and Bacteroidetes followed by Verrucomicrobia and Actinobacteria (Figure 3A). There were slight significant difference in seen in Actinobacteria & Proteobacteria in the overall community composition which might be due to inter-individual variations. We observed no difference between other subject characteristics such as birth mode (cesarean section or vaginal delivery) or antibiotic usage prior to the collection of samples (data not shown). Table 1 shows the average Firmicutes/Bacteroidetes ratio (F/B ratio) for each region ranging from 1.47 to 2.0 (Santo Andre, Natal, Porto Alegre, and Uberaba). F/B ratio has been shown by multiple studies to correlate with obesity and other metabolic diseases (31).

**Figure 4:**
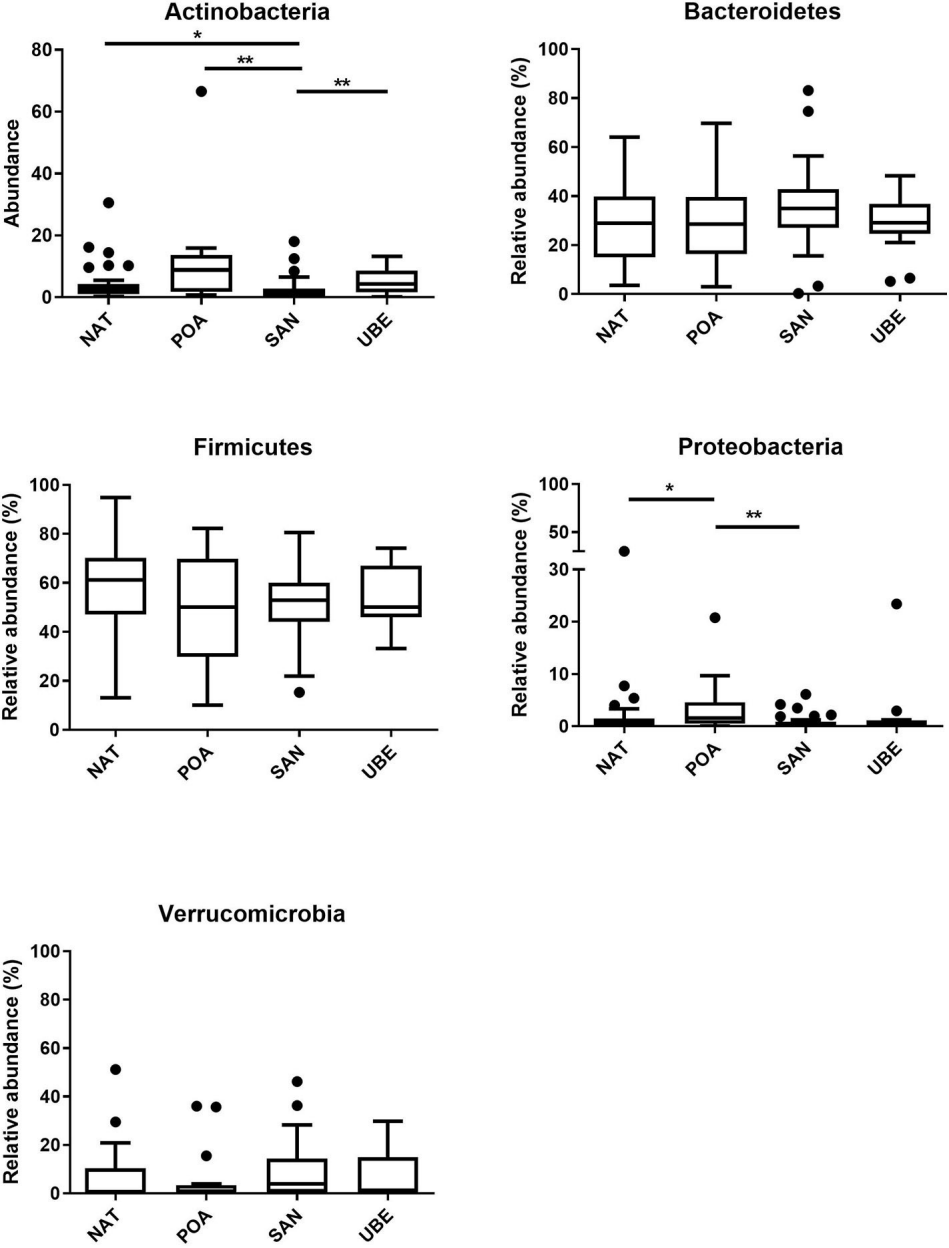
Comparison of various phyla across the four regions reveals significant differences in Actinobacteria and Proteobacteria. Where *p < 0.05, **p < 0.005.

Comparison of relative abundances at the bacterial family level revealed that the six most abundant bacterial families within the cohort were members of the phyla Firmicutes (*Ruminococcaceae* and *Lachnospiraceae*), Bacteroidetes (*Bacteroidaceae* and *Prevotellaceae*), Verrucomicrobium (*Verrucomicrobiaceae*), and Actinobacteria (*Bifidobacteriaceae*) (in order of relative abundance) (Figures 3, 4).

**Figure 5:**
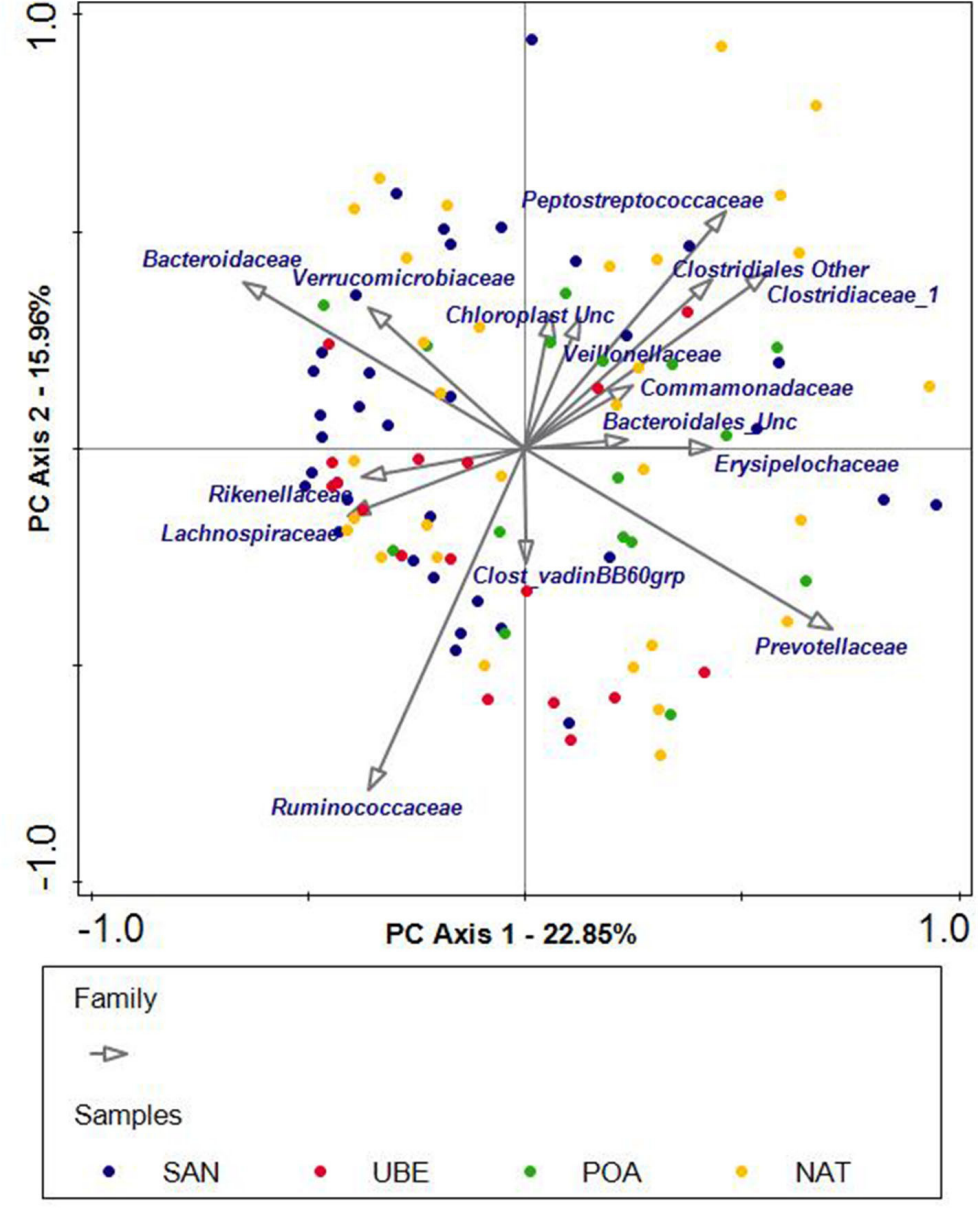
Unconstrained principal component analysis (PCA) of subjects grouped into four geographical regions: San Andre (SAN), Uberaba (UBE), Porto Alegre (POA), and Natal (NAT). Each dot represents one sample, and each color corresponds to a region. The PCA plot shows scattering of the samples across the quadrants. The arrows indicate the bacterial genera or families that potentially influence the scattering of the samples.

PCA was used to explore the taxonomic compositions of fecal samples. No clear clustering by the geographic origin was observed (Figure 5), which suggests that microbiota composition was not primarily driven by biogeographic factors.

### Microbiota Metabolite Functionality Indicators (Short-Chain Fatty Acid)

**Figure 6:**
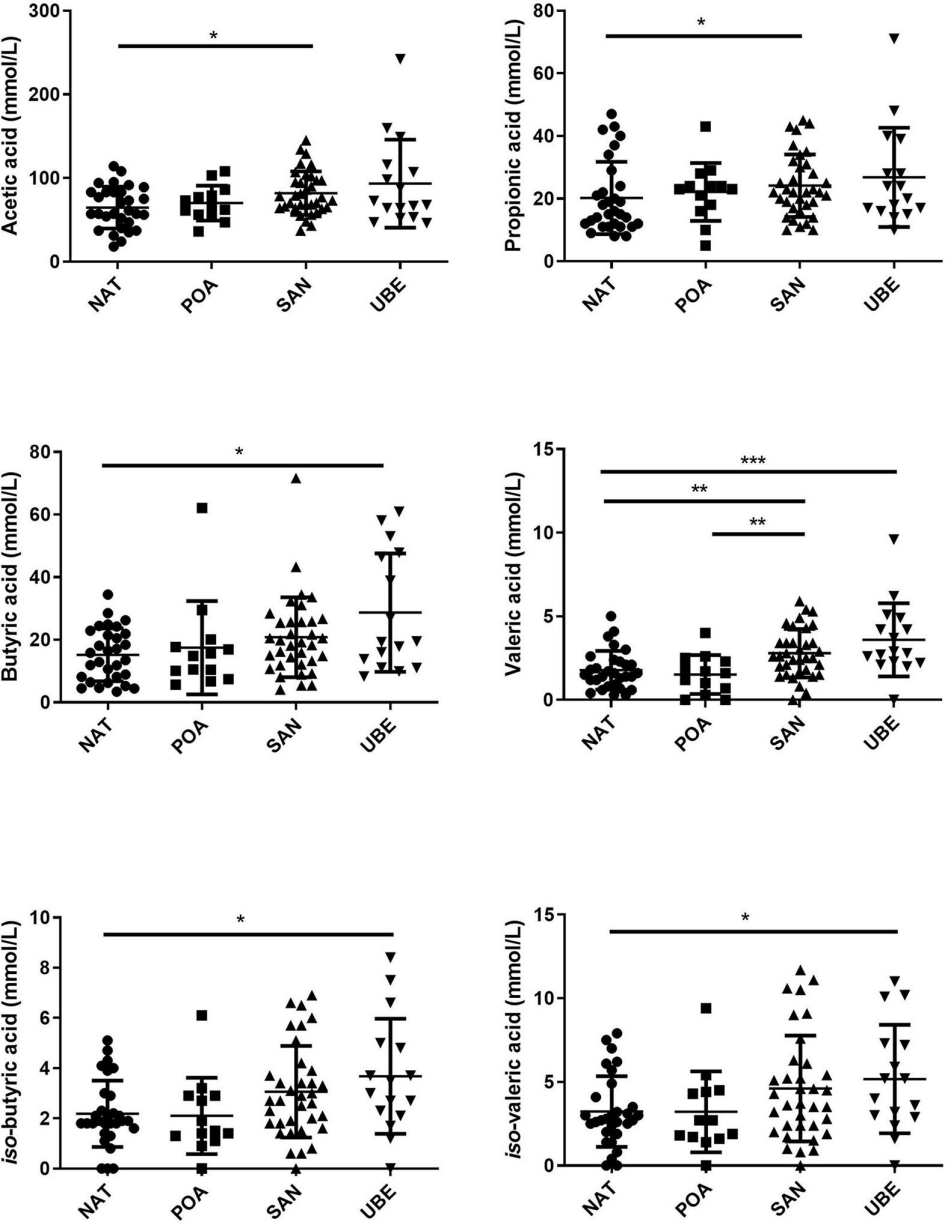
Comparison of short-chain fatty acids (SCFAs) across the geographical locations indicates that subject variation contributes to the significant differences observed in the different fatty acids. Where *p < 0.05, **p < 0.005, ***p < 0.0005.

The major detected metabolites were acetic, butyric, and propionic acids. There were small amounts of other valeric acid and the *iso*-forms of butyric and valeric acids, which are significantly different. It is also noted that Uberaba has the highest level of SCFA than the other three regions, although the profiles are very similar (see Figure 6 and Table 2).

**Table 2:** Average (minimum–maximum) fecal short chain fatty acid concentrations in mmol/kg of fecal material measured in subjects from the four sample sites.

**Average (Minimum–Maximum) concentration in mmol/kg**	**NAT**	**POA**	**SAN**	**UBE**
Acetic acid	64.66 (18–114)	70 (36–108)	81.86 (7–145)	93.3 (46–242)
Propionic acid	20.23 (8–47)	22.15 (5–43)	24.14 (10–45)	26.81 (10–71)
*iso*-butyric acid	2.43 (0.8–5.1)	2.28 (0.9–6.1)	3.15 (0.6–6.9)	3.93 (1.2–8.4)
Butyric acid	15.20 (3.4–34.4)	17.49 (5.6–62.1)	20.83 (4.1–71.6)	28.68 (8.3–60.9)
*iso*-valeric acid	3.46 (0.4–7.9)	3.48 (1.4–9.4)	4.74 (0.8–11.7)	5.52 (1.6–11)
Valeric acid	1.78 (0.3–5)	1.8 (0.3–4)	2.88 (0.4–5.9)	3.83 (2–9.6)
sIgA μg/ml homogenate	588.97,040	593.85 (80–1,880)	333.71 (20–1,460)	986.67 (30–5,740)

## Discussion

In this study, we have explored the microbiota composition of young children from four cities in Brazil located in three geographical regions. Although the locations are not near, the average microbiota composition was similar. Uberaba, based on demographics data (IBGE, Brazil 2019), has the most agriculture activity of the four regions. In our study, children from all centers are residing in urban areas and had access to different imported produce similarly for all day care centers. The Natal region is also characterized by exponential population growth. However, the degree of urbanization is less than that of the Southern parts of the country.

Bacterial diversity was found to be the greatest in children from Uberaba, indicated by highest alpha-diversity indices. The microbiota composition of these Brazilian children was dominated by Firmicutes and Bacteroidetes, which resembled more closely mature adult-like composition (31, 32). The F/B ratio has been extensively examined for human and mouse gut microbiota and has been linked to metabolic health by multiple studies utilizing this ratio to investigate obesity and age (31, 33). De Filippo et al. (23) compared fecal microbiota compositions of young children from a rural African village in Burkina Faso with fecal microbiota compositions of European children and proposed that children from less developed countries are characterized by lower F/B ratios (higher Bacteroidetes abundances), which may be an adaptation to a more polysaccharide-rich diet. The urbanized populations investigated in this study are also characterized by increased consumption of high-calorie food, which is low in nutritional value (junk food). This supports the hypotheses that energy-dense ultra-processed foods, a hallmark of the Western diet, indeed have pervasive effects on the microbiota development.

Other studies have reported positive correlations between F/B ratios and body mass index (BMI) and obesity (34, 35, 45). A study performed in Mexico reported that malnourished children have a higher F/B ratio than normal children of the same age. They have attributed this to the high amount of sugar and low-fiber diet that these children consumed (36). This may also be true for our study where children also consumed a lot of sweet juices and confectioneries (Figure 2). These habits might potentially increase children's predisposition to obesity in later life (25, 34, 37). In a further investigation, we found no significant interactions between BMI, dietary intake, and microbiota in our study. However, there are notable limitations to using only a 2-day recall questionnaire sampling diet in school and at home. These measurements might be biased due to caregiver's recollection and may not truly reflect a child's diet.

Interestingly, the Actinobacteria levels in the young children of this cohort are lower across all regions as compared with the abundances of this phylum observed in other microbiota studies. De Filippo et al. (23) reported high *Bifidobacterium* in European countries. In developed countries, *Bifidobacterium* has been documented to be an abundant and keystone member of the microbiota from infancy and all the way into toddlerhood (7, 9). Several Brazilian research groups have investigated *Bifidobacterium* spp. in various cities such as Porto Alegre (maternal-infant abundance) and Minas Gerais (1st months of life) (20, 38, 39). Another member of the phylum Actinobacteria, *Collinsella* was most abundant in children from Santo Andre. The genus *Collinsella* belongs to the family *Coriobacteriaceae* within the phylum Actinobacteria and has been positively correlated with insulin sensitivity and intestinal cholesterol absorption (35, 40). There is some evidence that *Collinsella* abundances depend on the dietary fiber intake (40, 41).

In line with the higher taxonomy diversity, the fecal SCFA profile also indicated that children from Uberaba have overall higher levels than children from the other three regions. This suggests that diet consumed by Uberaba children is richer in plant-based fibers, which facilitates microbial SCFA production. Fecal acetate, which is commonly associated with the activity of *Bifidobacterium* species, has been linked with decreased enteropathogenic infections by acidification of the gut (42). In our cohort, we observed low abundance of bifidobacterial species; this suggests the presence of other acetic acid-producing bacteria from the different genera. This suggests that the microbiota of these Brazilian children are different in this aspect than the children from the Amazonas of Venezuela, rural Malawi, and US metropolitan areas described Yatsunenko et al. (6), who report decreasing levels of *Bifidobacterium* with age.

A large-scale nutritional study across Brazil concluded that public schools have standardized food guidelines, which were the main factor driving food consumption to be similar (43). In the same study, household surveys showed a large population of children aged between 2 and 6 years who were not full time enrolled in the schools and required more careful assessment of their food intake and nutritional status. This study also highlighted that in several regions, consumption of processed sugary foods is high, even though nutrient levels are adequate.

To our knowledge, our study is one of the first to describe the gut microbiota composition, dietary intake, and function in young children across several cities in Brazil. We hypothesize that the microbiota of these Brazilian young children rapidly transited to a more adult-like microbiota profile unlike what has been reported for children of similar ages from either Western countries or “rural” areas in Africa and South America characterized by lower consumption of processed foods.

In conclusion, our study suggests that urban dietary factors play a larger role in influencing the gut microbiota composition, although they are separated geographically. It also illustrates the importance of considering the microbiota composition in diet, nutrition, and lifestyle recommendations and evaluating the impact of Westernization on health. This study is limited in that it only includes cross sections of these population at a single time point. A longitudinal sampling will be required to understand potential difference in microbiota of children from the same state with different socioeconomic status and to investigate how these environmental and lifestyle factors may contribute to later life health.

## Data Availability Statement

The datasets presented in this study can be found in online repositories. The names of the repository/repositories and accession number(s) can be found in the article/Supplementary Material.

## Ethics Statement

The studies involving human participants were reviewed and approved by CEP-FMABC, Comitê de Ética, Santo Andre. Written informed consent to participate in this study was provided by the participants' legal guardian/next of kin.

## Author Contributions

All authors have participated in the concept and design, analysis, and interpretation of data, drafting or revising of the manuscript. All authors have approved the manuscript and agree with the submission to Pediatrics.

## Conflict of Interest

The study design, data collection, analysis was carried out initially in Brazil with the participation of KB (full-time Danone Nutricia Brazil employee), MM, HM, ML, and VW. The study protocol is not directly related to any commercial product of the sponsor. CC (Danone Nutricia and Wageningen University), JK (Danone Nutricia and Wageningen University), and GR (Danone Nutricia) participated actively in the laboratory analysis of the intestinal microbiota and short chain fatty acids, interpretation of results, statistical analysis, and writing of the paper. MM, HM, ML, and VW declare that the research was conducted in the absence of any commercial or financial relationships that could be construed as a potential conflict of interest.
