# Supplementary material for: Gut Microbiota of Young Children Living in Four Brazilian Cities
Source: Front Pediatr. 2020 Dec 7;8:573815. doi: 10.3389/fped.2020.573815 (PMC7751462; doi:10.3389/fped.2020.573815)
Supplement: Supplementary file 1 [file Table_1.docx]

Supplementary Material

**Table 1.** Demographics of the mothers and children this study. Statistical test Mann-Whitney testing to ensure no bias in the data set

| *Maternal information* |  |  | | | *Geographical region* | | | |
| --- | --- | --- | --- | --- | --- | --- | --- | --- |
| Average (Min-Max) |  |  | | | **SAN** | **UBE** | **POA** | **NAT** |
| No. of participants |  |  | | | 35 | 17 | 16 | 31 |
| Age (in months) |  |  | | | 33.2 (22-45) | 29.2 (21-43) | 30 (18-49) | 32.5 (22-45) |
| Education (in Years) |  |  | | | 10.5 (3-16) | 10.2 (2-15) | 10.4 (6-14) | 9.7 (3-17) |
| Supplementation |  | Yes | | | 25 (71.43%) | 7 (41.18%) | 8 (50%) | 28 (90.32%) |
|  |  | No | | | 8 (22.86%) | 10 (58.82%) | 7 (43.75%) | 3 (9.68%) |
|  |  | NA | | | 2 (5.71%) | 0 (0%) | 1 (6.25%) | 0 (0%) |
| Employment |  | Employed | | | 31 (88.57%) | 8 (47.06%) | 13 (81.25%) | 15 (48.39%) |
|  |  | Unemployed | | | 1 (2.86%) | 4 (23.53%) | 1 (6.25%) | 11 (35.48%) |
|  |  | Retired | | | 1 (2.86%) | 0 (0%) | 0 (0%) | 0 (0%) |
|  |  | Others/NA | | | 2 (5.71%) | 3 (17.65%) | 2 (12.5%) | 2 (6.45%) |
| Complication (during pregnancy) |  | No | | | 30 (85.71%) | 9 (52.94%) | 15 (93.75%) | 13 (41.94%) |
|  |  | Yes | | | 4 (11.43%) | 8 (47.06%) | 1 (6.25%) | 17 (54.84%) |
|  |  | NA | 1 (2.86%) | | | 0 (0%) | 0 (0%) | 1 (3.23%) |
| *Child information* |  |  | | |  |  |  |  |
| Age (Months) |  |  | | 48.06 (29.16-61.36) | | 47.07 (38.85-58.07) | 33.20 (26.57-42.3) | 43.70 (28.74-57.29) |
| Gender |  | Male | | | 16 (45.71%) | 13 (76.47%) | 9 (56.25%) | 18 (58.06%) |
|  |  | Female | | | 19 (54.29%) | 4 (23.53%) | 7 (43.75%) | 13 (41.94%) |
| Height |  |  | | | 093.95 (76-102) | 94.23 (86.7-102.3) | 93.12 (83-104) | 84.10(72-93.5) |
| Weight |  |  | | | 15.7 (12.2-25.3) | 14.32 (12.35-17.95) | 14.49 (10.8-19.65) | 12.13 (8.10-16.60) |
| Mode of delivery |  | Vaginal | | | 12 (34.29%) | 8 (47.06%) | 10 (62.5%) | 12 (38.71%) |
|  |  | Caesarian | | | 23 (65.71%) | 9 (52.94%) | 5 (31.25%) | 19 (61.29%) |
| Any Illness (last 6 months) |  | No | | | 19 (54.29%) | 9 (52.94%) | 5 (31.25%) | 6 (19.35%) |
|  |  | Yes | | | 16 (45.71%) | 8 (47.06%) | 11 (68.75%) | 25 (80.65%) |
| Antibiotics use (last 6 months) |  | No | | | 12 (34.29%) | 10 (58.82%) | 9 (56.25%) | 7 (22.58%) |
|  |  | Yes | | | 23 (65.71%) | 7 (41.18%) | 7 (43.75%) | 24 (77.42%) |

*NA- results were not collected from subject
